# How late is too late for treatment?

**DOI:** 10.7554/eLife.86117

**Published:** 2023-02-07

**Authors:** Lawrence T Reiter

**Affiliations:** 1 Department of Neurology, Department of Pediatrics, Department of Anatomy and Neurobiology, College of Medicine, University of Tennessee Health Science Center Memphis United States

**Keywords:** ASO, RNA therapeutics, neurodevelopmental disorder, EEG, Mouse

## Abstract

Experiments on mice suggest that an approach called antisense oligonucleotide therapy may be able to treat some symptoms of Angelman syndrome, including problems with epilepsy and sleep.

**Related research article** Lee D, Chen W, Kaku HN, Zhuo X, Chao ES, Soriano A, Kuncheria A, Flores S, Kim JH, Rivera A, Rigo F, Jafar-nejad P, Beaudet AL, Caudill MS, Xue M. 2023. Antisense oligonucleotide therapy rescues disturbed brain rhythms and sleep in juvenile and adult mouse models of Angelman syndrome. *eLife* **12**:e81892. doi: 10.7554/eLife.81892.

An approach called antisense oligonucleotide (ASO) therapy has ushered in a new age in genetic medicine. ASO therapy works by introducing a short strand of RNA that binds to specific messenger RNA (mRNA) molecules in the host, and thus prevents the mRNA from being translated. Clinical trials are currently under way to see if ASO therapy will work for various neurodevelopmental disorders, including Dravet syndrome (an epilepsy disorder), spinal muscular atrophy (a neuromuscular condition) and Batten’s disease (a devastating lysosomal storage disorder; Hill and Meisler, 2021).

Angelman syndrome is a neurodevelopmental disorder that is considered an ideal candidate for ASO therapy. Symptoms appear very early in childhood and include learning disabilities, abnormally happy demeanor, epilepsy, and difficulty controlling motor function, particularly while walking (Dagli et al., 1993). Children with Angelman syndrome also suffer from sleep problems. It has previously been shown, using cellular and animal models, that the regulation of a single gene, *UBE3A*, in the nervous system leads to the major features of Angelman syndrome (Kishino et al., 1997; Matsuura et al., 1997; Sutcliffe et al., 1997). *UBE3A* is found on chromosome 15, and most cases of Angelman syndrome are the result of a large deletion in the maternal copy of this chromosome. This means that most individuals have a working – but silent – paternal copy of *UBE3A* on chromosome 15. However, this copy is silenced by an antisense transcript which interferes with the expression of the paternal *UBE3A*.

Using ASO therapy to interfere with the antisense transcript – and thus allowing the intact copy of *UBE3A* to be expressed – is a promising approach for the treatment of Angelman syndrome. However, some scientists remain skeptical about the potential for ASO therapy to treat neurodevelopmental disorders, and several questions remain regarding how these treatments will work. For example, when does it become too late in human development to reactivate a missing gene in the nervous system? Are there neurogenetic diseases that can be rescued in adulthood? And, if so, what features of the disease can be treated with ASO therapies?

Extensive research has focused on answering these questions by reactivating the paternal copy *UBE3A* in a commonly used mouse model for Angelman syndrome. One goal of these studies has been to determine which symptoms can be reduced or eliminated. Another goal, which may be more challenging to achieve, is to establish when the gene should be reactivated during development in order to achieve the desired effect.

In 2018, researchers at the Erasmus Medical Center in Rotterdam published a set of behaviors that can be used to assess phenotypes for motor performance, repetitive behavior, anxiety, and seizure susceptibility using *Ube3a* maternal deficient mice (Sonzogni et al., 2018). These behaviors provide a framework to test the effectiveness of drugs (or ASOs) that reactivate the silent paternal copy of the gene. However, there are not many studies that dig deeper into the cognitive issues, sleep or epilepsy-related brain activity (as measured with EEG) that are known to be affected in this mouse model. Now, in eLife, Mingshan Xue and colleagues from Baylor College of Medicine and Ionis Pharmaceuticals – including Dongwon Lee, Wu Chen, Heet Naresh Kaku and Xinming Zhuo as first authors – report on the use of an ASO to rescue the characteristic EEG pattern and disordered sleep observed in a mouse model of Angelman syndrome (Lee et al., 2023).

First, Lee et al. designed a new Angelman syndrome mouse model that is less ‘leaky’ than the model used by other labs in previous studies – that is, a model where *Ube3a* expression from the maternal chromosome was more completely blocked. Then they injected the mice with an ASO against the *Ube3a* antisense transcript to see if the expression of the Ube3a protein could be rescued from the paternal chromosome. The results showed that, after injecting the mice with the ASO, the levels of Ube3a protein increased in multiple regions of the brain, including the cortex, the hippocampus and the hypothalamus, which controls sleep. Importantly, *Ube3a* expression was rescued in both juvenile and adult animals, which had previously been challenging.

Next, Lee et al. showed that the electrical activity in the brain of these mice is significantly rescued by injection of this ASO, in both juvenile and adult animals. They were also able to rescue the low level of rapid eye movement (REM) sleep observed in individuals with Angelman syndrome, with animals getting an almost normal amount of REM sleep six weeks after injection with the ASO.

The findings of Lee et al. illustrate that it may be possible to treat some aspects of Angelman syndrome after birth, and even into adulthood, using ASO therapeutics. This challenges the current view of what symptoms of Angelman syndrome are treatable, and at what age. While the delivery of ASOs to the brain is still a struggle, the latest results are encouraging for potential treatments for Angelman syndrome, and perhaps other neurodevelopmental disorders thought to be untreatable after birth.

## References

[bib1] Dagli AI, Mathews J, Williams CA (1993). Angelman syndrome. GeneReviews.

[bib2] Hill SF, Meisler MH (2021). Antisense oligonucleotide therapy for neurodevelopmental disorders. Developmental Neuroscience.

[bib3] Kishino T, Lalande M, Wagstaff J (1997). UBE3A/E6-AP mutations cause Angelman syndrome. Nature Genetics.

[bib4] Lee D, Chen W, Kaku HN, Zhuo X, Chao ES, Soriano A, Kuncheria A, Flores S, Kim JH, Rivera A, Rigo F, Jafar-nejad P, Beaudet AL, Caudill MS, Xue M (2023). Antisense oligonucleotide therapy rescues disturbed brain rhythms and sleep in juvenile and adult mouse models of Angelman syndrome. eLife.

[bib5] Matsuura T, Sutcliffe JS, Fang P, Galjaard RJ, Jiang YH, Benton CS, Rommens JM, Beaudet AL (1997). De novo truncating mutations in E6-AP ubiquitin-protein ligase gene (UBE3A) in Angelman syndrome. Nature Genetics.

[bib6] Sonzogni M, Wallaard I, Santos SS, Kingma J, du Mee D, van Woerden GM, Elgersma Y (2018). A behavioral test battery for mouse models of Angelman syndrome: A powerful tool for testing drugs and novel *Ube3a* mutants. Molecular Autism.

[bib7] Sutcliffe JS, Jiang YH, Galijaard RJ, Matsuura T, Fang P, Kubota T, Christian SL, Bressler J, Cattanach B, Ledbetter DH, Beaudet AL (1997). The E6-AP ubiquitin-protein ligase (UBE3A) gene is localized within a narrowed Angelman syndrome critical region. Genome Research.

